# Supplementary material for: Structural signatures in EPR3 define a unique class of plant carbohydrate receptors
Source: Nat Commun. 2020 Jul 30;11:3797. doi: 10.1038/s41467-020-17568-9 (PMC7392887; doi:10.1038/s41467-020-17568-9)
Supplement: Supplementary file 1 — Supplementary Information [file 41467_2020_17568_MOESM1_ESM.pdf]

SUPPLEMENTARY INFORMATION

Structural signatures in EPR3 define a unique class of plant carbohydrate receptors

Wong et al.

Supplementary Table 1 Data collection and refinement statistics

	EPR3
**Data collection**	
Space group	P 1 21 1
Cell dimensions	
a, *b*, *c* (Å)	59.82, 36.08, 72.66
α, β, γ (°)	90.00, 93.92, 90.00
Resolution range (Å)	47.7-1.87 (1.94-1.87)*
R_{meas} (%)	9.8 (94.6)
CC1/2	0.99 (0.82)
$I / \sigma I$	15.72 (1.82)
Completeness (%)	96.9 (84.2)
Redundancy	6.3 (4.7)
**Refinement**	
No. unique reflections	25173 (2151)
Reflections in R_{free} set	2000
R_{work} / R_{free} (%)	16.3 / 21.0
No. residues	302
No. atoms	
Protein	2362
Glycosylation + small solutes	42
Water	170
B-factors (Å^2)	
Protein	39.8
Glycosylation + small solutes	83.9
Water	44.4
R.m.s. deviations	
Bond lengths (Å)	0.015
Bond angles (°)	1.17

Data was collected from one crystal.
*Values in parentheses are for highest-resolution shell.

Supplementary Table 2 SAXS data collection and scattering-derived parameters

Protein	EPR3	EPR3 + R7A EPS
**Data collection**		
Beamline	PETRA III P12	PETRA III P12
Wavelength (nm)	1.24	1.24
s range (nm^{-1})	0.120 - 3.340	0.106 - 3.063
Exposure time (s)	0.045	0.045
Concentration range (mg/ml) (data shown for 6.8)	0.6 - 22	0.6 - 22
Ligand concentration (M)	N/A	0.001
Temperature (°C)	20	20
**Structural parameters**		
Rg (nm) from Guinier	2.29 ± 0.2	2.22 ± 0.1
Rg (nm) from P(r) plot	2.28 ± 0.07	2.02 ± 0.04
D_{max} (nm)	7.5	7.1
Porod volume estimate (Å^3)	56.7	52.5
Estimated molecular mass from Porod volume (kDa)	29.0	26.5
Estimated molecular mass from forward scattering I(0) (kDa)	24.7	24.7
Calculated molecular mass from amino acid sequence (kDa)*	23.6	23.6
Estimated mass of glycosylated EPR3 from SDS-PAGE (kDa)	25-35	25-35
**Software employed**		
Primary data reduction	Primus	
Ab initio analysis	BioXtas RAW	
Rigid body modeling	DAMMIN/DAMMIF	
Visualization	colores/PyMOL	

* The molecular mass was calculated without considering the contributions from glycosylation.

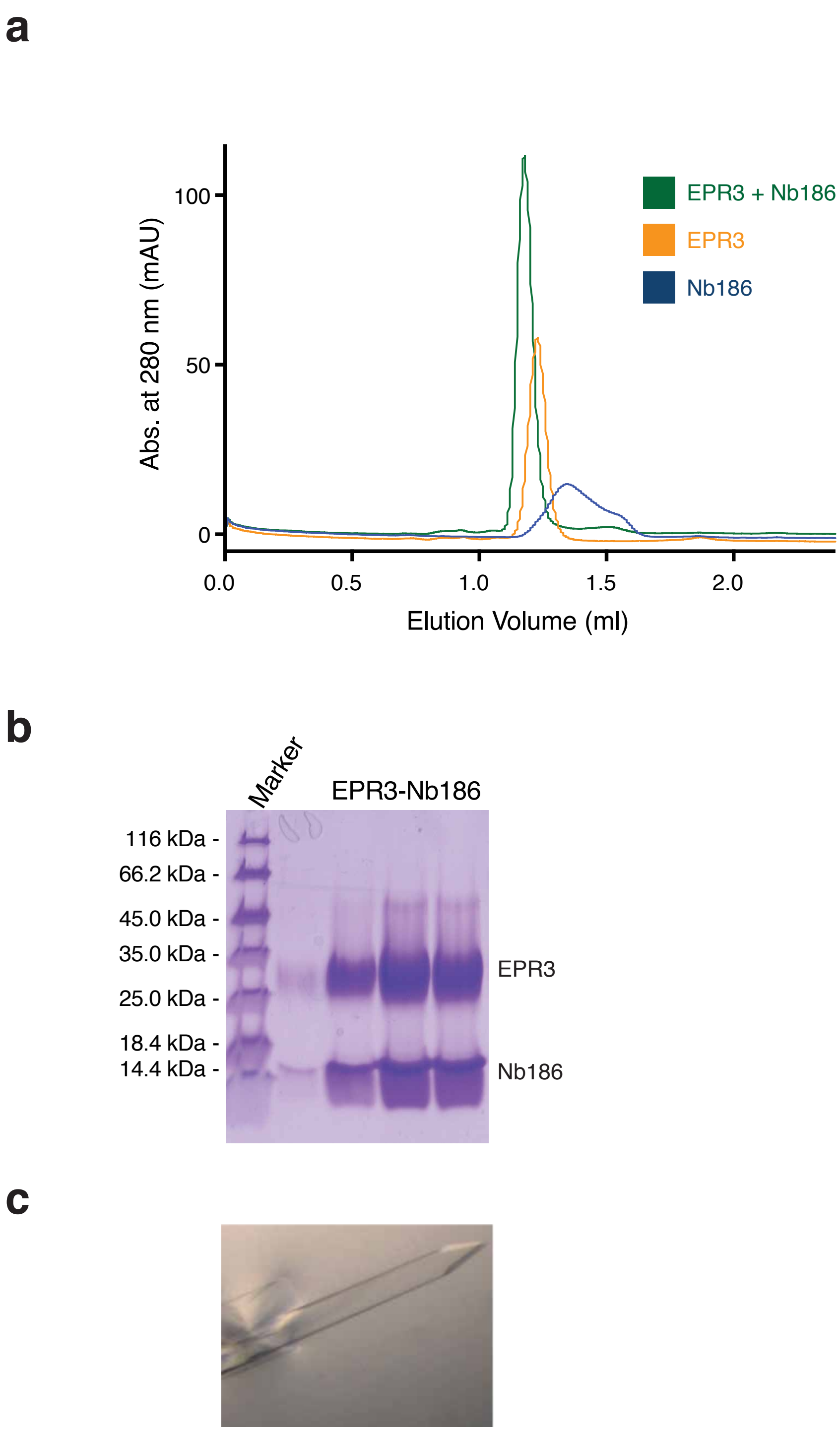

Supplementary Fig. 1 | Purification of the EPR3-Nb186 complex. **a**, Overlay of chromatograms from SEC experiments with EPR3, Nb186 and the EPR3-Nb186 complex. **b**, SDS-PAGE of the EPR3-Nb186 complex used for crystallization and **c**, a representative image of a single EPR3-Nb186 crystal.

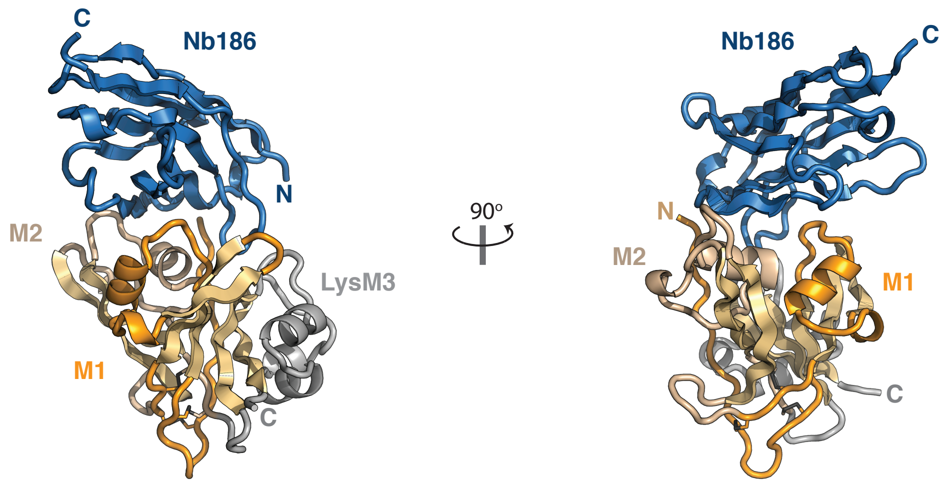

Supplementary Fig. 2 | The EPR3-Nb186 structure. Cartoon representation of the EPR3-Nb186 complex crystal structure with Nb186 coloured in blue and EPR3 coloured as in Fig. 1.

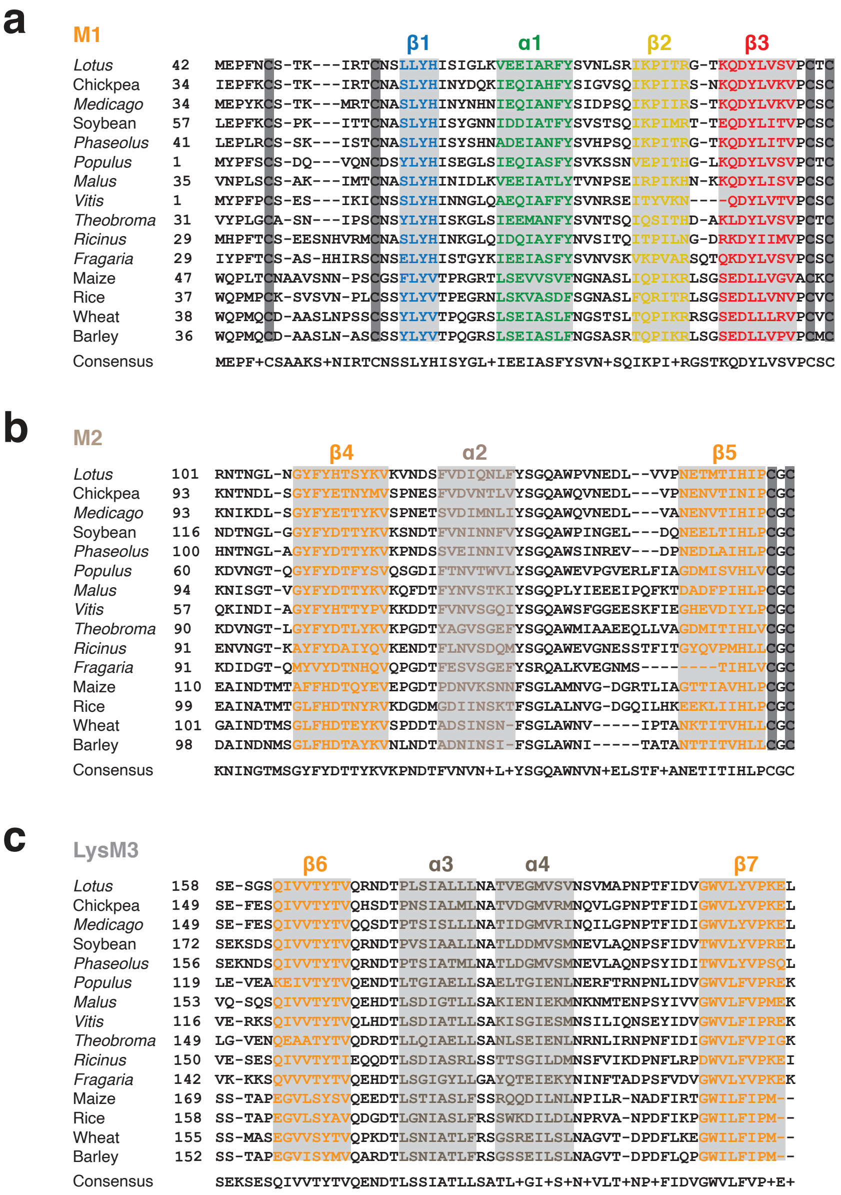

Supplementary Fig. 3 | Sequence alignment of EPR3 homologues. Sequence alignment of the extracellular domain of EPR3 homologues from dicots (legumes and non-legumes) and monocots showing the conserved secondary structure arrangement of **a**, M1 (βαββ fold) **b**, M2 (βαβ fold) and **c**, LysM3 (βααβ fold). *Lotus* (BAI79269.1), Chickpea (XP_004489790.1), *Medicago* (XP_003613165.1), Soybean (XP_003517716.1), *Phaseolus* (XP_007157313.1), *Populus* (XP_002322185.1), *Malus* (XP_008340354.1), *Vitis* (XP_002272814.2), *Theobroma* (XP_007036352.1), *Ricinus* (XP_002527912.1), *Fragaria* (XP_004300916.1), Maize (XP_008657477.1), Rice (XP_015628733.1), Wheat (CDM80098.1) and Barley (MLOC_5489.2).

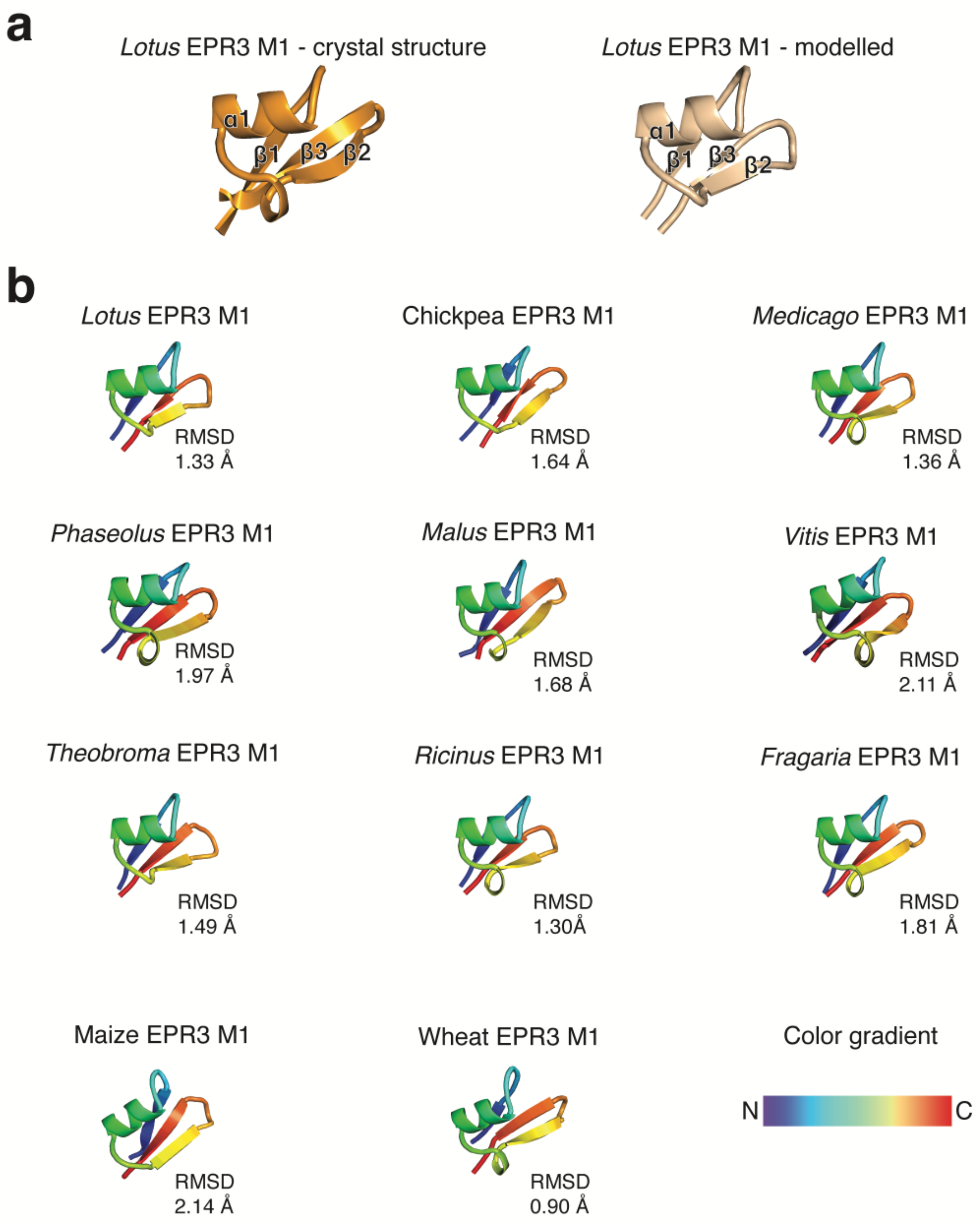

Supplementary Fig. 4 | Structural modelling of M1 from EPR3 homologues. **a**, Side-by-side comparison of the EPR3 crystal structure of M1 and an *ab-initio* atomic-level force field model of EPR3 M1. **b**, Modelling of M1 from EPR3 homologues reveals the same overall βαββ arrangement. The molecular fit (RMSD) in Å to the crystal structure of EPR3-M1 is denoted.

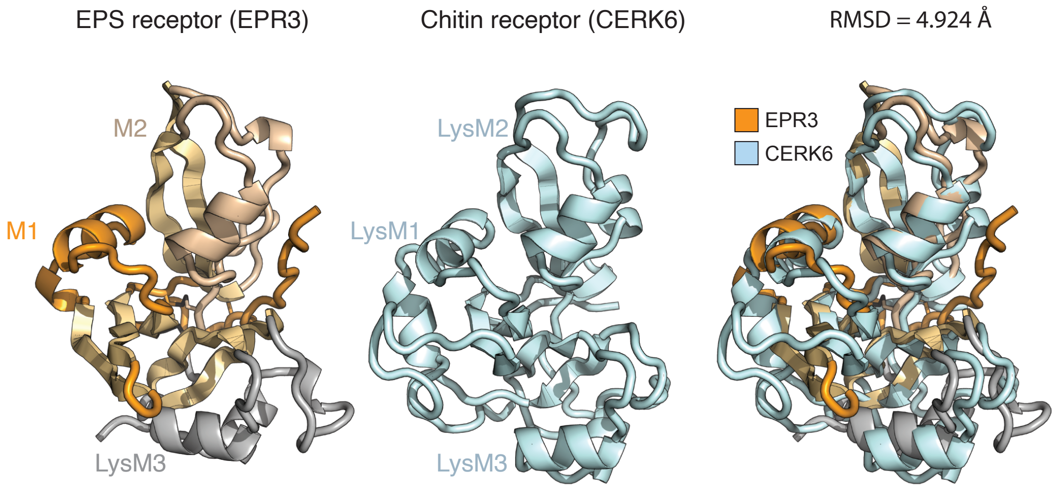

Supplementary Fig. 5 | Structural comparison of plant receptors. Cartoon representations of EPS (*Lotus* EPR3) and chitin (*Lotus* CERK6) receptors coloured as indicated. The root mean square deviation (RMSD) of the structural superposition is given in Å (Rigid-body superimposition of 112 equivalent Cα atoms).

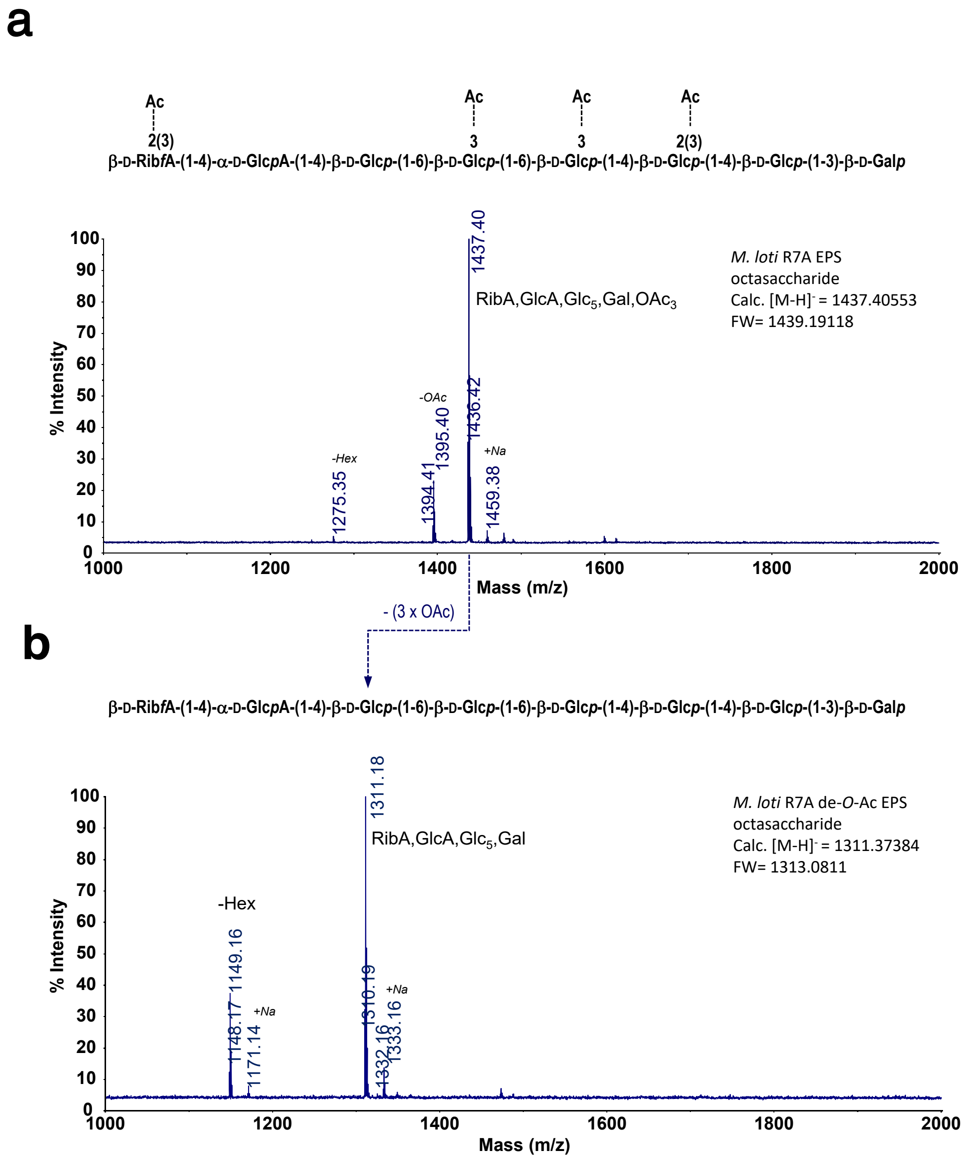
a
Ac
2(3)
Ac
3
Ac
3
Ac
2(3)
β-D-RibfA-(1-4)-α-D-GlcpA-(1-4)-β-D-Glcp-(1-6)-β-D-Glcp-(1-6)-β-D-Glcp-(1-4)-β-D-Glcp-(1-4)-β-D-Glcp-(1-3)-β-D-Galp
% Intensity
1437.40
RibA,GlcA,Glc5,Gal,OAc3
M. loti R7A EPS
octasaccharide
Calc. [M-H]- = 1437.40553
FW= 1439.19118
-OAc
1436.42
1395.40
1394.41
-Hex
1275.35
+Na
1459.38
Mass (m/z)
- (3 x OAc)
b
β-D-RibfA-(1-4)-α-D-GlcpA-(1-4)-β-D-Glcp-(1-6)-β-D-Glcp-(1-6)-β-D-Glcp-(1-4)-β-D-Glcp-(1-4)-β-D-Glcp-(1-3)-β-D-Galp
% Intensity
1311.18
RibA,GlcA,Glc5,Gal
M. loti R7A de-O-Ac EPS
octasaccharide
Calc. [M-H]- = 1311.37384
FW= 1313.0811
-Hex
1149.16
1148.17
+Na
1171.14
1310.19
+Na
1332.16
1333.16
Mass (m/z)

c

β-D-GlcNAc*p*-(1-4)-β-D-GlcNAc*p*-(1-4)-β-D-GlcNAc*p*-(1-4)-β-D-GlcNAc*p*-(1-4)-β-D-GlcNAc*p*-(1-4)-β-D-GlcNAc*p*

d

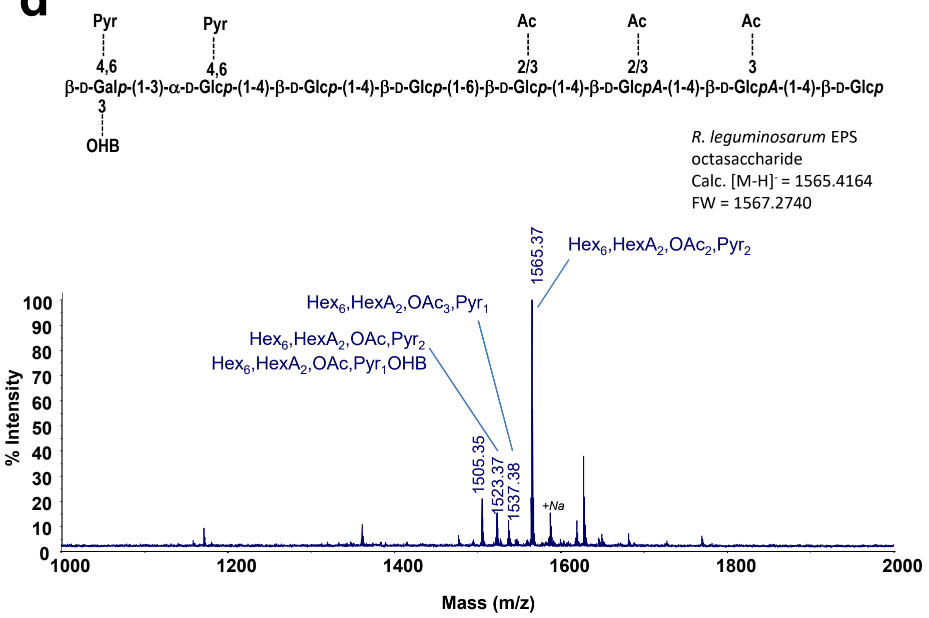

e

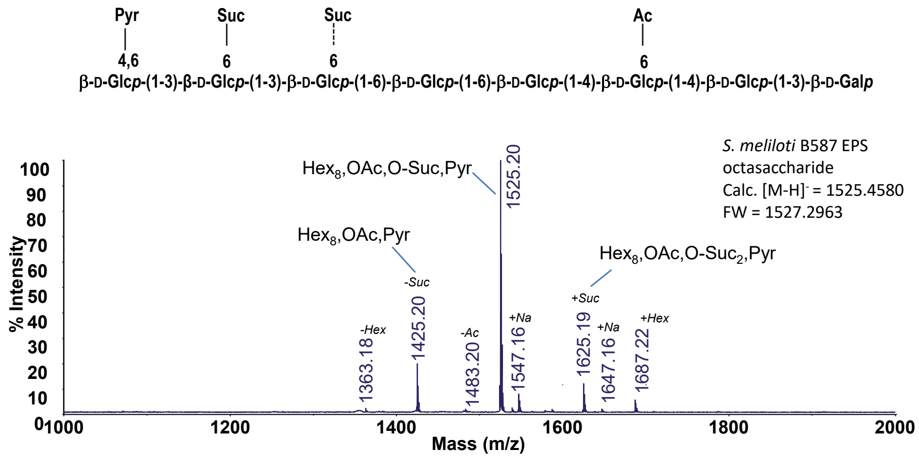

Supplementary Fig. 6 | Carbohydrate ligands used in this study. The proposed structures of the EPS ligands are shown together with their corresponding MALDI-TOF MS spectrum. **a**, R7A EPS **b**, R7A deOAc-EPS **c**, Chitohexose (CO6) control **d**, *R. leguminosarum* EPS and **e**, *S. meliloti* EPS.

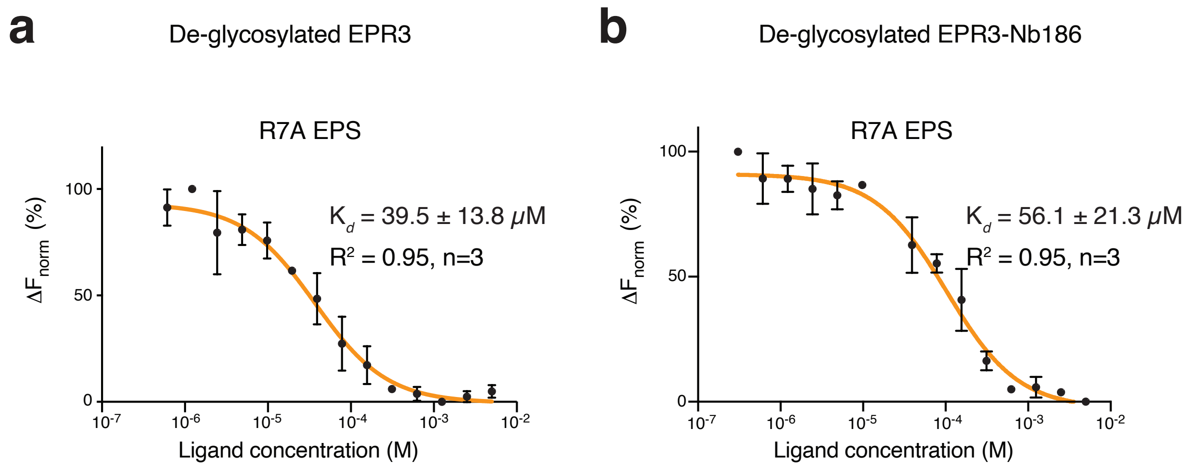

Supplementary Fig. 7 | Binding experiments with de-glycosylated EPR3 and de-glycosylated EPR3-Nb186 complex. **a**, MST binding experiment of de-glycosylated EPR3 to R7A EPS. **b** MST binding experiment of de-glycosylated EPR3-Nb186 complex to R7A EPS. The equilibrium dissociation constants (K_d) in the 95% confidence interval, goodness of fit (R^2), and number of independent protein preparations used for the measurements (n) are reported.

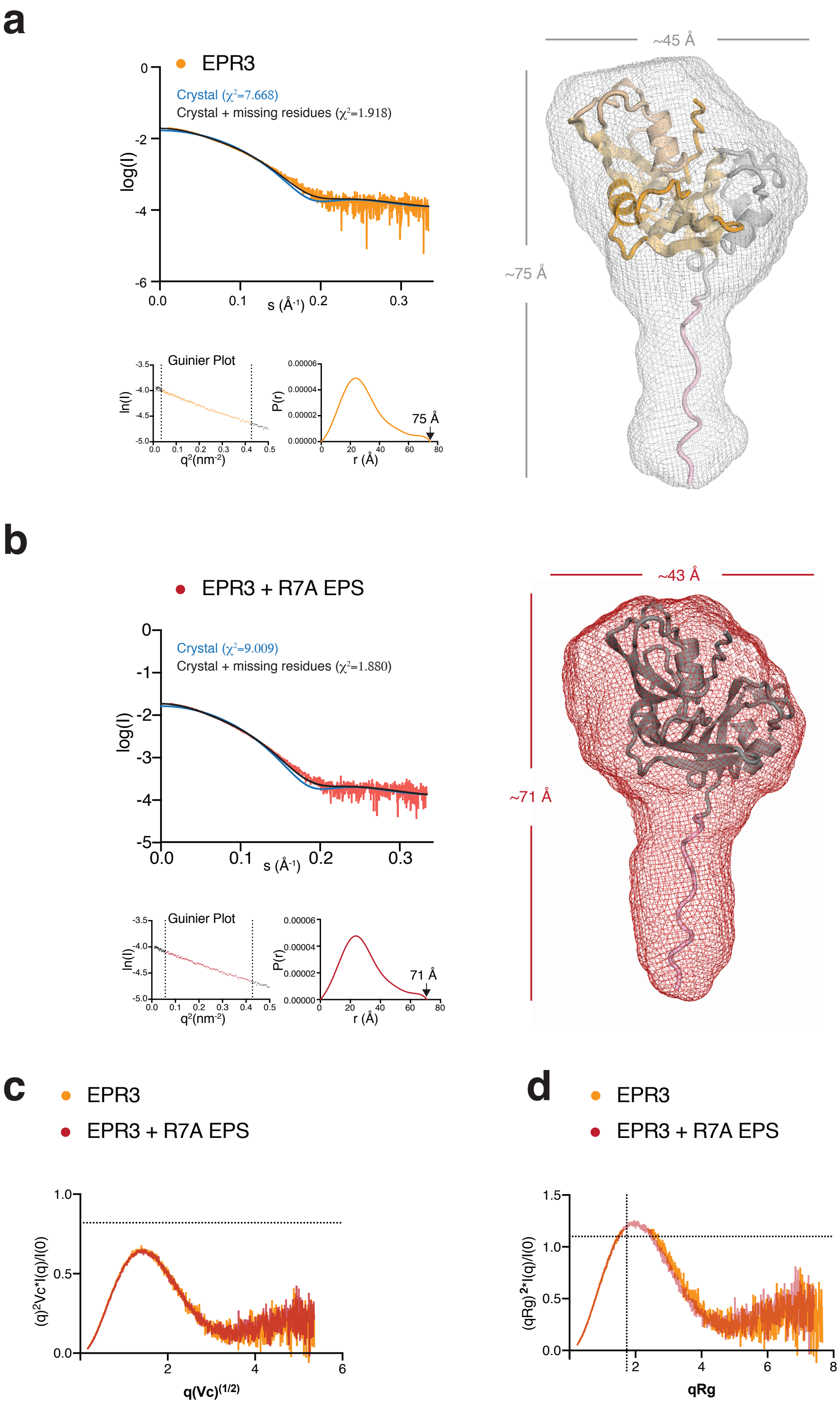
a
EPR3
Crystal (χ²=7.668)
Crystal + missing residues (χ²=1.918)
log(I)
s (Å⁻¹)
Guinier Plot
ln(I)
q²(nm⁻²)
P(r)
r (Å)
75 Å
~45 Å
~75 Å
b
EPR3 + R7A EPS
Crystal (χ²=9.009)
Crystal + missing residues (χ²=1.880)
log(I)
s (Å⁻¹)
Guinier Plot
ln(I)
q²(nm⁻²)
P(r)
r (Å)
71 Å
~43 Å
~71 Å
c
EPR3
EPR3 + R7A EPS
(q)²Vc*I(q)/I(0)
q(Vc)^(1/2)
d
EPR3
EPR3 + R7A EPS
(qRg)²*I(q)/I(0)
qRg

Supplementary Fig. 8 | SAXS data, fits and models. SAXS analysis of EPR3 showing scattering curve with model fit for EPR3 in the **a**, absence of ligand and **b**, in the presence of R7A EPS ligand. Guinier plots and P(r) distance distribution plot with D_{max} are shown together with docking of EPR3 into the SAXS envelope. EPR3 has an extended stem-like structure in both the absence and presence of ligand. The overall dimensions are shown in angstrom (Å). **c, d** Dimensionless Kratky plots of EPR3 with and without R7A EPS. **c,** Vc-based with the volume of an ideal sphere (dotted line, y = 0.82) and **d**, Rg-based with indicated Guinier-Kratky point ($\sqrt{3}/1.1$, dotted lines). Addition of the ligand does not induce changes in either flexibility or globularity of EPR3.
